# Comprehensive analysis of immune infiltration and gene expression for predicting survival in patients with sarcomas

**DOI:** 10.18632/aging.202229

**Published:** 2020-12-09

**Authors:** Hongmin Chen, Yijiang Song, Chuangzhong Deng, Yanyang Xu, Huaiyuan Xu, Xiaojun Zhu, Guohui Song, Qinglian Tang, Jinchang Lu, Jin Wang

**Affiliations:** 1 Department of Musculoskeletal Oncology, Sun Yat-Sen University Cancer Center, Guangzhou 510060, Guangdong, P. R. China; 2 State Key Laboratory of Oncology in Southern China, Collaborative Innovation Center of Cancer Medicine, Guangzhou 510060, P. R. China

**Keywords:** sarcoma, tumor-infiltrating immune cells, immune checkpoint

## Abstract

Tumor microenvironments are strongly related to tumor development, and immune-infiltrating cells and immune-related molecules are potential prognostic markers. However, the shortcomings of traditional measurement methods limit the accurate evaluation of various components in tumor microenvironments. With the rapid advancement of Next-Generation RNA Sequencing technology, dedicated and in-depth analyses of immune filtration within the tumor microenvironment has been achieved. In this study, we combined the bioinformatics analysis methods ESTIMATE, CIBERSORT, and ssGSEA to characterize the immune infiltration of sarcomas and to identify specific immunomodulators of different pathological subtypes. We further extracted a functional enrichment of significant immune-related genes related to improved prognosis, including NR1H3, VAMP5, GIMAP2, GBP2, HLA-E and CRIP1. Overall, the immune microenvironment is an important prognostic determinant of sarcomas and may be a potential resource for developing effective immunotherapy.

## INTRODUCTION

Sarcomas, a broad family of mesenchymal malignancies, exhibit remarkable histologic diversity [1]. Soft tissue sarcomas are malignancies caused by extra-skeletal connective tissue (including the peripheral nervous system) and consist of more than 80 pathological types [2]. Sarcomas are rare; an estimated 13,130 sarcomas are diagnosed each year in the United States, accounting for approximately 1% of 1,806,590 new malignancies [3]. Many are highly aggressive and account for a high proportion of cancer mortality in young adults (in SEER data, seer.cancer.gov). Soft tissue sarcomas occur with great frequency in patients with specific mutations, such as the APC mutation and TP53 mutation [1, 4, 5].

The tumor microenvironment (TME) is composed of various cell types (e.g., endothelial cells, fibroblasts and immune cells) and extracellular components (e.g., extracellular matrix, cytokines and growth factors) [6]. In the TME, immune cells and stromal cells are the two main types of non-tumor components and are indicated to be of great significance in the prognosis evaluation [7, 8]. A previous study reported that immune surveillance may play an important role in the progression of sarcomas [9]. The response rates were less than 20% with either pembrolizumab monotherapy or with combination ipilimumab/nivolumab therapy [10, 11]. However, effective responses to immune checkpoint blockade therapy have been observed in specific subtypes, including high-grade undifferentiated pleomorphic sarcomas, clear cell sarcomas and dedifferentiated liposarcomas [10–13]. Different immune characteristics may have a significant impact on efficacy. Therefore, it is urgent to explore the features of sarcoma immune microenvironment cells and immune checkpoints, which may provide potential prognostic factors and treatment targets for clinical therapy.

Immune microenvironment-related bioinformatic algorithms have been applied to cervical cancer [14], breast cancer [15], and gliomas [16], showing the effectiveness of the algorithms [17]. However, the practicality of immune scores for addressing sarcomas has not been studied. ESTIMATE (Estimation of STromal and Immune cells in MAlignant Tumor tissues using Expression data), an algorithm to calculate immune and stromal scores, predicts the infiltration of non-cancer components [18]. The Cancer Genome Atlas sarcoma (TCGA SARC) database is available to understand potential correlations between gene set profiles and overall survival from malignancies [19]. To better understand the proportions of immune cells in the TME, we used CIBERSORT (Cell type Identification By Estimating Relative Subsets Of RNA Transcripts) deconvolution software and ssGSEA (single sample Gene Set Enrichment Analysis) to determine the relative proportions of several distinct leukocyte cell types in sarcomas from microarray gene expression data of sarcoma patients [20, 21].

Thus, we first obtained a list of genes that predict good outcomes in sarcoma patients by making use of the ESTIMATE, CIBERSORT and ssGSEA algorithms. Finally, we validated these genes in an independent sarcoma cohort from the Gene Expression Omnibus (GEO) dataset GSE17679.

## RESULTS

### Immune scores and stromal scores of sarcoma subtypes

**Figure 1:**
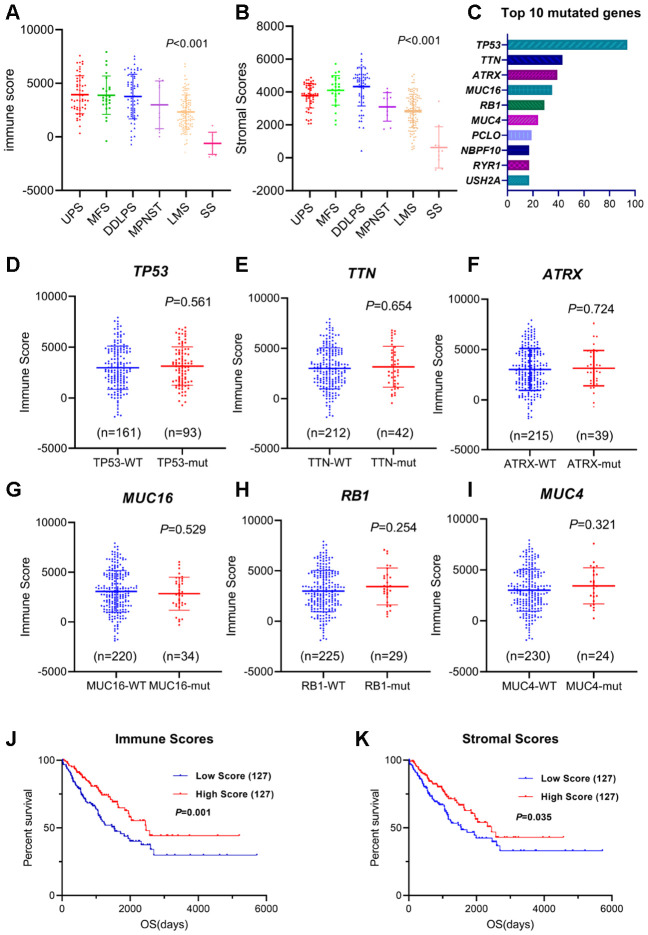
**Immune/stromal scores of different sarcoma subtypes and survival.** (**A**, **B**) Immune scores and stromal scores for different sarcoma subtypes. One-way ANOVA was applied. (**C**) Top 10 somatic mutation of TCGA sarcoma cohort. (**D**–**I**) Distribution of immune scores for *TP53*, *TTN*, *ATRX*, *MUC16*, *RB1* and *MUC4* mutant/wildtype sarcoma cases. Student's t test was applied. (**J**, **K**) The Kaplan-Meier survival curves of immune scores and stromal scores. Sarcoma cases were divided into high- and low-score groups based on the median. The sample number for each group was listed in brackets. Statistical significance was determined using the log-rank test.

The gene expression profiles and clinical information of all 254 sarcoma patients with an initial pathologic diagnosis and their overall survival were downloaded from the database of TCGA. All sarcoma cases with complete gene expression data and clinical information in TCGA were included in our analysis. Among all cases, the pathological diagnoses included 58 (22.8%) cases of dedifferentiated liposarcoma (DDLPS), 103 (40.6%) cases of leiomyosarcoma (LMS), 25 (9.8%) cases of myxofibrosarcoma (MFS), 9 (3.5%) cases of malignant peripheral nerve sheath tumor (MPNST), 10 (3.9%) cases of synovial sarcoma (SS) and 49 (19.3%) cases of undifferentiated pleomorphic sarcoma (UPS). According to the ESTIMATE algorithm, the average immune scores of the UPS cases were ranked highest among all six pathological subtypes, followed by those of MFS, DDLPS, MPNST and LMS. The SS cases had the lowest immune scores (Figure 1A, *P*<0.001, one-way ANOVA). Meanwhile, the stromal scores of DDLPS, MFS and UPS were ranked the highest among all subtypes, followed by MPNST and LMS. The stromal scores of SS cases were also the lowest (Figure 1B, *P*<0.001, one-way ANOVA). These results showed the patterns of microenvironmental variance across these pathological subtypes.

Based on the TCGA somatic mutation database, TP53, TTN, ATRX, MUC16, RB1 and MUC4 make up the highest frequency of somatic mutations in sarcomas (Figure 1C). We explored the relationship between the mutations of these genes and the immune scores and found that the mutation status was not correlated with immune scores (Figure 1D–1I, Student's t test), indicating that the main type of mutation in sarcoma might not cause specific changes in immune infiltration.

To examine the correlations between overall survival and immune scores or stromal scores, we divided the cases into two halves (high scores and low scores). A Kaplan-Meier survival curve showed that the survival time of the high-immune-score group was longer than that of the low-score group (Figure 1J, *P*=0.001). Consistently, patients in the low-stromal-score group were significantly worse off in their overall survival times compared to the high-score group (Figure 1K, *P*=0.035).

### Gene expression profiles of immune/stromal scores in sarcomas

**Figure 2:**
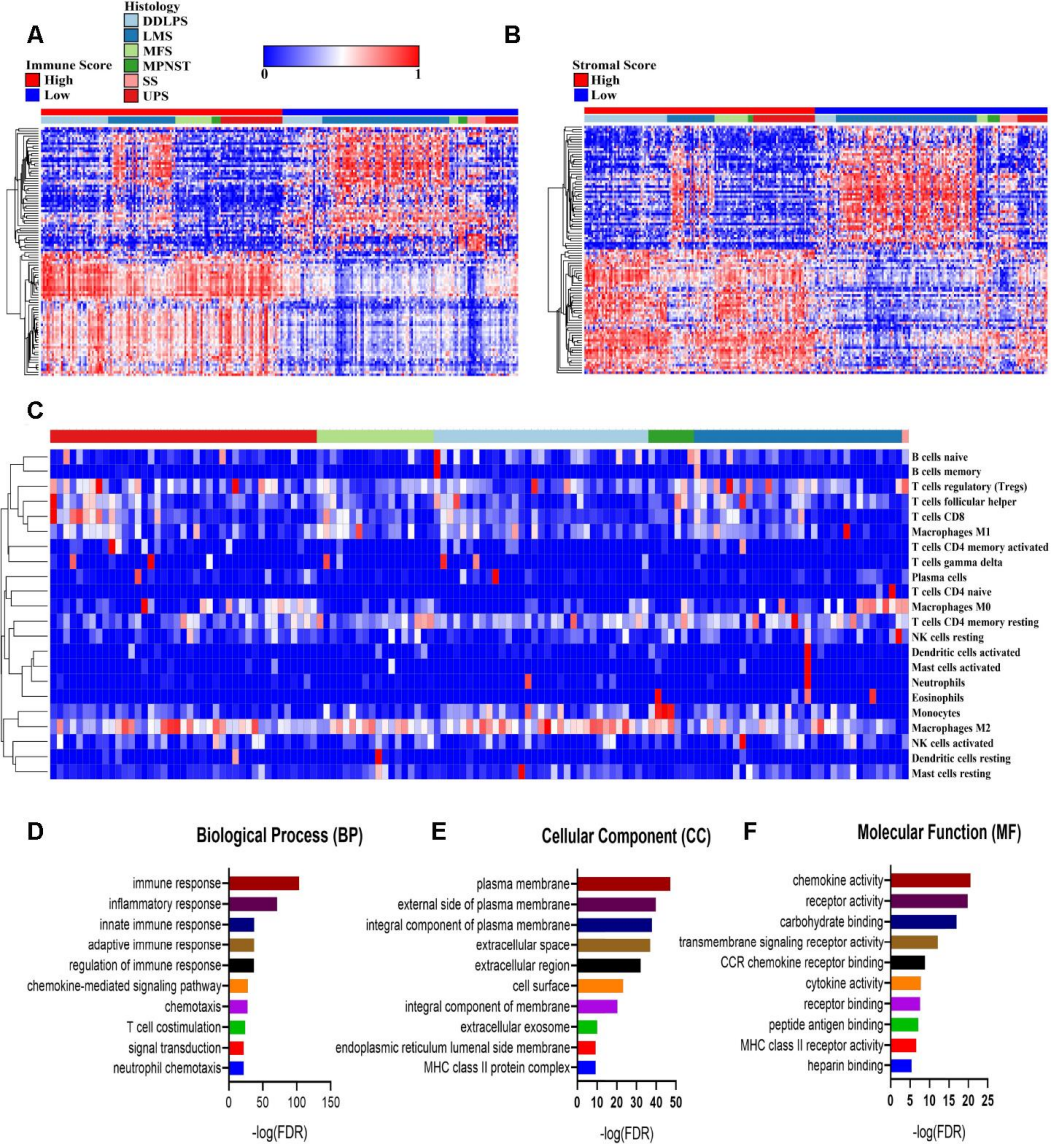
**Comparisons of differentially expressed genes (DEGs) with immune scores and stromal scores.** (**A**, **B**) The 100 top differentially expressed genes of high- and low- immune scores and stromal scores. The cohort was divided into high- and low-score groups based on the median. (**C**) The proportion of immune cells estimated by the CIBERSORT algorithm with outputs in which *P*<0.05 in 119 sarcoma samples. (**D**–**F**) Enrichment analysis of differentially expressed genes between groups with high and low immune scores. DDLPS: dedifferentiated liposarcoma, LMS: leiomyosarcoma, MFS: myxofibrosarcoma, MPNST: malignant peripheral nerve sheath tumor, SS: synovial sarcoma and UPS: undifferentiated pleomorphic sarcoma.

To reveal the differential gene expressions in the immune score/stromal scores, we compared the RNA-seq data of 254 sarcoma patients obtained from the database of TCGA. The differentially expressed genes (DEGs) in the immune/stromal groups with high or low scores have different characteristics. In terms of the immune-score groups, 1,396 genes were upregulated and 949 genes were downregulated in the high-score group compared with the low-score group (fold change>2, *P*<0.05). Similarly, for the stromal-score groups, the high-score group had 1,533 upregulated genes and 969 downregulated genes (fold change>2, *P*<0.05) compared with the low-score group. Figure 2A, 2B plots the heatmap of the top 100 DEGs with high or low scores. In addition, the CIBERSORT deconvolution algorithm was used to calculate the proportions of 22 types of immune cells in sarcoma samples (Figure 2C and Supplementary Figure 1A). The type 2 macrophages in sarcomas made up the largest composition of all immune cells in the TME of sarcomas (except synovial sarcoma). The results indicate a suppressive TME in sarcomas.

To further reveal the biological processes, cellular components and molecular functions of the DEGs, we performed a functional enrichment analysis of the genes that were upregulated in the high-immune-score group that fold change>3. The top gene ontology (GO) terms identified included immune (including innate and adaptive) response, plasma membrane and chemokine activities. (Figure 2D–2F).

### Genes for survival prediction in sarcomas

**Figure 3:**
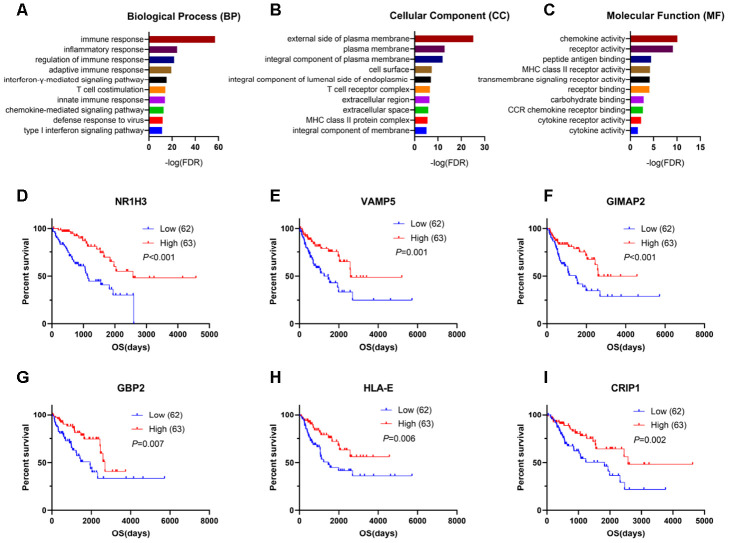
**Genes for survival prediction among sarcoma patients.** (**A**–**C**) Enrichment analysis of good survival-related genes among sarcoma patients. (**D**–**I**) The Kaplan-Meier survival curves for sarcoma patients further separated into the high and low expression groups based on the quartiles of the NR1H3, VAMP5, GIMAP2, GBP2, HLA-E and CRIP1 mRNA levels, separately.

To explore the potential roles of individual immune-related DEGs on the overall survival of sarcoma patients, we used univariate Cox regression analyses and Kaplan-Meier survival curves. Among the DEGs upregulated in the high-immune-score group, a total of 528 DEGs were significantly related to good overall survival, as determined by univariate Cox regression analysis. Functional enrichment analysis showed a strong association between these genes and the immune response as well. The top GO terms mainly included immune responses, plasma membrane, T cell receptor complex, chemokine activity and receptor binding (Figure 3A–3C, Supplementary Table 1).

After performing log-rank tests in the TCGA SARC cohort and validating these good-prognosis-related genes with the GEO dataset GSE17679, we extracted information indicating that NR1H3, VAMP5, GIMAP2, GBP2, HLA-E and CRIP1 were highly expressed in the immune microenvironment and were most significantly associated with predicting good outcomes in sarcoma patients (Figure 3D–3I, Supplementary Figure 2).

### Protein-protein interactions among prognosis-related genes

To better understand the interplay among the identified DEGs, we obtained protein-protein interaction (PPI) networks using the STRING tool. The networks were mainly made up of six modules, which included 514 nodes and 1,635 edges.

**Figure 4:**
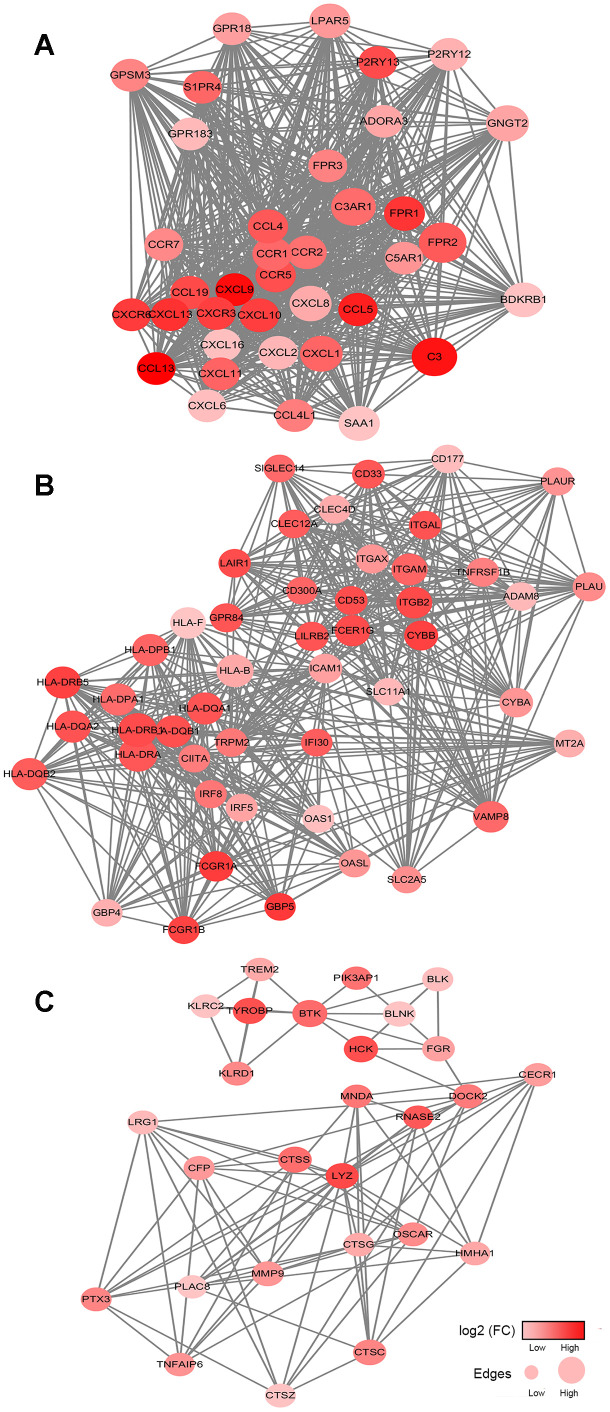
**Network analysis results of the top 3 protein-protein interaction networks for DEGs significantly associated with overall survival.** (**A**–**C**) The cell chemotaxis network, immune response network and leukocyte mediated immunity network. The gradient color scale indicates the log value of expression fold change (log2 (FC)), and node sizes reflect the number of interactions identified for each protein. FC: fold change.

The top three significant modules were selected for further study. In the cell chemotaxis network (Figure 4A), CCL13, CXCL9, C3, CCL5 and CXCL13 were the core nodes since they had the highest expressions. For the immune response network (Figure 4B), most nodes were correlated with the response to interferon-gamma, including ICAM1, HLA-B, HLA-F, and HLA-D family. For the leukocyte mediated immunity network (Figure 4C), BTK, TYROBP, CTSS, CTSC and LYZ had higher degree values and were enriched in leukocyte degranulation and myeloid leukocyte activity.

### The prognostic model of immune checkpoint modulators in sarcomas

**Figure 5:**
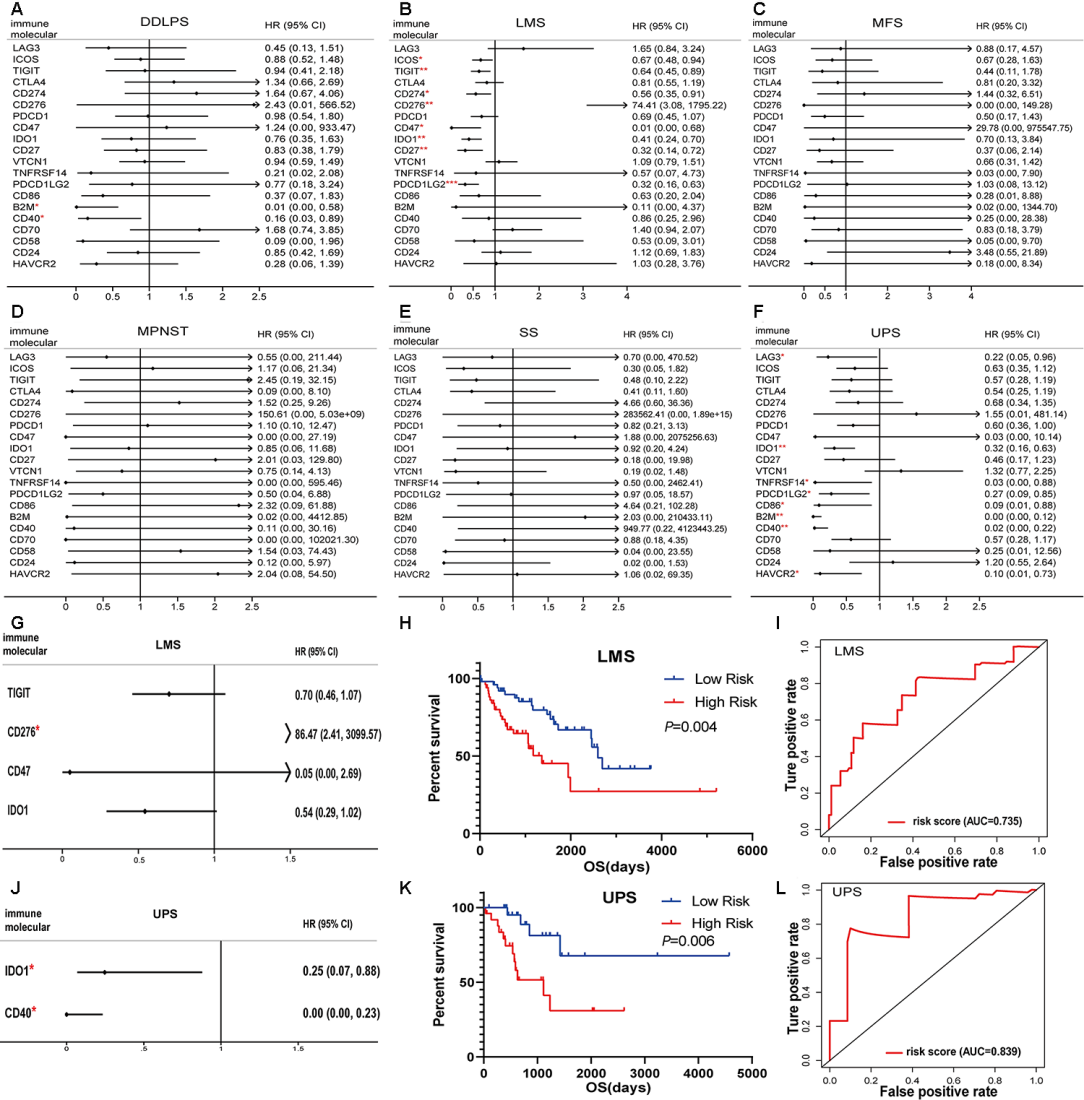
**Immunomodulators significantly associated with prognosis in sarcoma.** Forest plots of univariate Cox-regression analysis for 20 immunomodulators in the (**A**) DDLPS, (**B**) LMS, (**C**) MFS, (**D**) MPNST, (**E**) SS and (**F**) UPS. Forest plot of multivariate Cox analysis for the immunomodulators model in the (**G**) LMS and (**J**) UPS. The hazard ratio with 95% CI and *P*-values were illustrated in the figure (* p < 0.05, ** p < 0.01, *** p < 0.001). The immunomodulator model significantly predicts OS in (H) LMS and (**K**) UPS, and the high- and low- risk groups were divided based on the median. Receiver-operating characteristic (ROC) analysis of one-year survival prediction by the immunomodulator model in (**I**) LMS and (**L**) UPS. AUC: area under the curve, DDLPS: dedifferentiated liposarcoma, LMS: leiomyosarcoma, MFS: myxofibrosarcoma, MPNST: malignant peripheral nerve sheath tumor, SS: synovial sarcoma, UPS: undifferentiated pleomorphic sarcoma and HR: hazard ratio.

In sarcoma patients, the efficacy of emerging immune checkpoint blockade therapies (such as PD1 inhibitors) has been linked to the expression of immune checkpoint signaling molecules and immune cell infiltration fractions [22]. We performed a univariate Cox regression analysis of 20 immunomodulators on six pathological subtypes of sarcoma patients. B2M and CD40 were associated with the good prognostic value among DDLPS patients (Figure 5A). ICOS, TIGIT, CD274, CD276, CD47, IDO1, CD27 and PDCD1LG2 predicted good outcomes for LMS (Figure 5B). No immunomodulators had prognostic value for MFS, MPNST and SS (Figure 5C–5E). Moreover, LAG3, IDO1, TNFRSF14, PDCD1LG2, CD86, B2M, CD40 and HAVCR2 were associated with favorable outcomes in UPS patients (Figure 5F).

The prognostic factors identified by univariate Cox analysis were further analyzed in a multivariate Cox model for LMS and UPS. The results indicated that CD276 was an independent risk factor (Figure 5G). According to the immune risk score, the immunomodulator model for LMS was established based on the combination of TIGIT, CD276, CD47 and IDO1 after the stepwise selection, and the prognosis index was calculated as follows:

Risk score = -0.3529 × exp_TIGIT_ + 4.4598 × exp_CD276_ − 3.0041 × exp_CD47_ − 0.6090 × exp_IDO1_

where exp indicates the log2(mRNA expression).

Similarly, a predictive model for UPS was also established according to the regression coefficients. The results indicated that IDO1 and CD40 could be independent risk factors (Figure 5J). The model had the power to calculate the prognostic risk score by the following formula:

Risk score = -1.3995 × exp_IDO1_ − 8.8754 × exp_CD40_

where exp indicates the log2(mRNA expression).

Patients with a high-risk score in the immunomodulator model tended to have unfavorable outcomes. The Kaplan-Meier curves showed that a high-risk score was significantly associated with poor prognoses in LMS and UPS (Figure 5H, 5K). The area under the curve (AUC) values of the receiver operating characteristic (ROC) were 0.735 and 0.839, respectively, which indicated that these models had great value in estimating patient survival (Figure 5I, 5L).

### Prognostic value of infiltration immune cells in sarcoma

**Figure 6:**
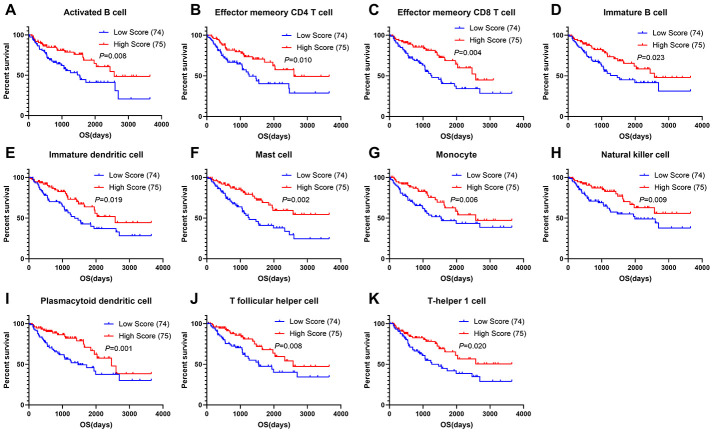
**Correlations of immune cell scores and overall survival in sarcoma.** (**A**–**K**) Infiltrating immune cells significantly associated with improved prognosis. The high- and low-score groups were divided based on the top 30% and the bottom 30% infiltrating scores calculated by ssGSEA algorithm, respectively)

To assess the predictive value of immune cells in the TME, we examined the relationship between overall survival and different immune cell distribution patterns. For each patient, the relative infiltration scores of the 28 immune cell subpopulations were calculated using ssGSEA (Supplementary Figure 3). As shown in Figure 6, Kaplan-Meier survival analyses showed that activated B cells, effector memory CD4^+^ T cells, effector memory CD8^+^ T cells, immature B cells, immature dendritic cells, mast cells, monocyte, natural killer cells, plasmacytoid dendritic cells, T follicular helper cells and Th1 cells were significantly associated with improved prognoses.

## DISCUSSION

There is increasing awareness that sarcomas are one of the most challenging obstacles when trying to promote immune responses to cancer. The current understanding of the sarcoma TME is limited, but previous studies have demonstrated that sarcomas might be cold tumors and that suppressor cells, including tumor-associated macrophages and myeloid-derived suppressor cells (MDSC), constitute the immune infiltration rather than exhausted CD8^+^ T cells [23, 24]. Moreover, PD-L1 expression is relatively low in sarcomas, and the mutation burden is also relatively low [19, 25]. To improve the therapeutic treatments for sarcomas, research on immunomodulators and the infiltration of immune cells would be the appropriate future efforts.

First, by comparing the DEGs in the two groups of samples with high and low immune scores, we found that the high-score group, with better prognoses, was enriched in immune-related pathways such as T cell costimulation, immune receptor binding, and antigen processing via MHC-I and cytokine activity. After performing validation in both TCGA dataset and GEO dataset GSE17679, we extracted a group of immune-related genes, including NR1H3, VAMP5, GIMAP2, GBP2, HLA-E and CRIP1 that were most significant to predict good outcomes in sarcoma patients. These immune-related genes were rarely studied in tumors but has certain therapeutic value. NR1H3 promotes the monocyte to macrophage transition and prolongs the length of survival in nasopharyngeal carcinoma [26]. GTPases of Immunity-Associated Proteins (GIMAPs) are related to the regulation of apoptosis in lymphocytes [27]. Guanylate-binding proteins (GBPs) mediate a broad scope of innate immune functions in response to several pro-inflammatory cytokines [28]. Similarly, VAMP5, HLA-E and CRIP1 have been confirmed to be involved in immune regulation [29, 30]. Extracting the potential of these molecules in anti-tumor response seems to be necessary for future work.

Besides, we extracted six protein-protein interaction modules, all of which are related to immune responses. MHC molecules present antigens for cells in the adaptive immune system, including cytotoxic CD8+ T cells. Loss or downregulation of class I MHC was observed in a large percentage of soft tissue sarcomas patients [31–33]. For sarcoma patients with low MHC-I expression, their overall survival and event-free survival significantly worsened compared with those in the high-expression group [34]. Meanwhile, many cytokines have been employed for several sarcoma subtypes with intriguing results. Tumor necrosis factor α and melphalan (TNF-ILP) have benefited patients with locally advanced primary and recurrent extremity sarcoma in multimodal treatments [35]. Finding antigen-presenting molecules and cytokines related to favorable prognoses will help to design new clinical trials for sarcomas.

We further assessed the prognostic value of immunomodulators in sarcomas. Immune checkpoint inhibition is an encouraging approach for immunotherapy, namely the removal of the "brakes" of the immune system [36]. Building on the limited clinical success of immune checkpoint agents for sarcomas, immunomodulators are expected to treat cancer effectively and durably [11, 37–40]. Tawbi et al. reported that in a single-arm phase II study of pembrolizumab among 40 patients with soft-tissue sarcoma, seven patients (18%) showed an objective response, including four of 10 patients (40%) with UPS, two of 10 patients (20%) with liposarcoma, and one of 10 patients (10%) with SS [11]. The initial results of the CTLA-4 blockade ipilimumab and dasatinib study showed no response per the Response Evaluation Criteria in Solid Tumors (RECIST) or Immune-Related Response Criteria [39]. Thus, extracting new specific checkpoints has become necessary. The checkpoint molecules analyzed in this study represented immune response signals, including costimulatory, coinhibitory and situation-dependent signals. Two immune checkpoint modulators (e.g., B2M and CD40) were associated with good prognostic value for DDLPS, seven modulators (e.g., ICOS, TIGIT, CD274, CD276, CD47, IDO1, CD27 and PDCD1LG2) were identified as prognostic molecules for LMS, and eight modulators were significantly correlated with UPS outcomes in the univariate Cox regression model. In the immunomodulator risk score model, the combination of TIGIT, CD276, CD47 and IDO1 had a certain value in estimating survival of LMS patients, while IDO1 and CD40 had a higher predictive value for UPS, as indicated by the AUC. These immune response signals may be future targets in sarcoma treatment to further rev up or release the brakes of the immune system [41].

Finally, we evaluated the prognostic value of infiltrating immune cells in sarcoma. The results showed that 11 kinds of highly infiltrating immune cells were predictors of longer overall survival, most of which have been confirmed in previous studies. After B cell activation by T helper cells initiates the humoral immune response to most protein antigens, T follicular helper (Tfh) cells provide signals to B cells, including cytokines and cell surface ligands, to direct isotype switching and activate germinal center formation, somatic hypermutation, and affinity maturation [42–44]. Tfh cells regulate humoral immunity and cell-mediated anti-tumor responses and improve overall survival [45, 46]. Evaluating the functions of Tfh cells and exploring their interactions with B cells and T helper cells in sarcoma therefore represents a potential therapeutic strategy. Our results were in accordance with previous research, showing that Tfh cells, T helper cells and B cells infiltration predicts good prognoses in several cancers.

In conclusion, this study revealed distinct immune infiltration patterns and indicated that immunomodulators are essential determinants of sarcoma prognoses. Moreover, we extracted a list of tumor microenvironment related genes that could be useful for outlining the prognoses of sarcoma patients. The number of cohorts included in the study was small. Larger scale sequencing data will be tested for future work. The evolution of this comprehensive investigation will lead to the elucidation of the immunological mechanisms that affect sarcoma progression and the development of immunotherapeutic sarcoma trials.

## MATERIALS AND METHODS

### Database and differentially expressed genes analysis

The Level 3 RNA sequencing (RNAseq) data, somatic mutation data and clinical information of 254 sarcoma patients were downloaded from The Cancer Genome Atlas (TCGA) hub by the University of California, Santa Cruz, Xena browser (https://xenabrowser.net/datapages/). TCGA RNA sequencing data show the gene-level transcription estimates, as in log2(x + 1) transformed RSEM normalized counts. 64 cases from the Gene Expression Omnibus (GEO) database GSE17679 (https://www.ncbi.nlm.nih.gov/geo) were enrolled as a validation set. RNA expressions data for GSE17679 were using Affymetrix Human Genome U133 Plus 2.0 Array. The pan-cancer analysis was performed on the Gene Expression Profiling Interactive Analysis 2 (GEPIA2) webserver (http://gepia2.cancer-pku.cn/), which integrated RNA expression and clinical data from 33 TCGA cohorts. Differentially expressed genes (DEGs) were obtained using the R package edgeR [47]. Fold change>2 and *P*<0.05 were set as the cut-offs to screen for DEGs.

### Immune infiltration algorithm

To infer the fractions of stromal and immune cells in tumor samples, we applied the ESTIMATE algorithm to calculate the immune and stromal scores [18]. In addition, a deconvolution approach, the CIBERSORT algorithm [20], was introduced to estimate the fractions of 22 immune cell types. After 100 permutations of calculations, samples with outputs in which *P*<0.05 were considered to be available for further analysis. Finally, the resulting CIBERSORT values were defined as the immune cell infiltration fractions of each sample. Moreover, ssGSEA [21] was used to quantify the relative infiltration scores of 28 immune cell types in the TME. Feature gene panels for each immune cell type were collected from a recent publication [48]. The enrichment score in ssGSEA represented the relative abundance of each immune cell type.

### Functional enrichment analysis and PPI network

For functional analysis, gene ontology (GO) analyses were conducted via DAVID (The Database for Annotation, Visualization and Integrated Discovery) [49]. False discovery rates (FDR)<0.05 were considered significant. The protein-protein interaction (PPI) networks were retrieved from the STRING database [50].

The DEGs list was used for protein-protein interaction (PPI) analysis in Cytoscape (v3.7.1, National Resource for Network Biology, https://cytoscape.org/). Samples with outputs with interaction scores>0.9 were considered in further study. Only individual networks with 10 or more nodes were included for Molecular COmplex DEtection (MCODE), which could explore clusters based on subnetwork module analysis to find densely connected components.

### Statistical analysis

The Kaplan–Meier survival curves with the log-rank test were used to estimate the correlation between DEGs or immune cell types and overall survival. The Mantel-Cox test was performed in the pan-cancer survival analysis for patients from 33 disease cohorts. Multivariate analyses applied stepwise selection and Cox proportional hazard regression model. Statistical analyses were performed using R version 3.6.0. For all tests, *P*<0.05 was considered to be statistically significant.

### Code availability

R and other custom codes for analyzing data are available upon request to the authors.

## Supplementary Material

Supplementary Figures

Supplementary Table 1
