# Supplementary material for: Comprehensive analysis of immune infiltration and gene expression for predicting survival in patients with sarcomas
Source: Aging (Albany NY). 2020 Dec 9;13(2):2168–83. doi: 10.18632/aging.202229 (PMC7880383; doi:10.18632/aging.202229)
Supplement: Supplementary Figures [file aging-13-202229-s001.pdf]

SUPPLEMENTARY FIGURES

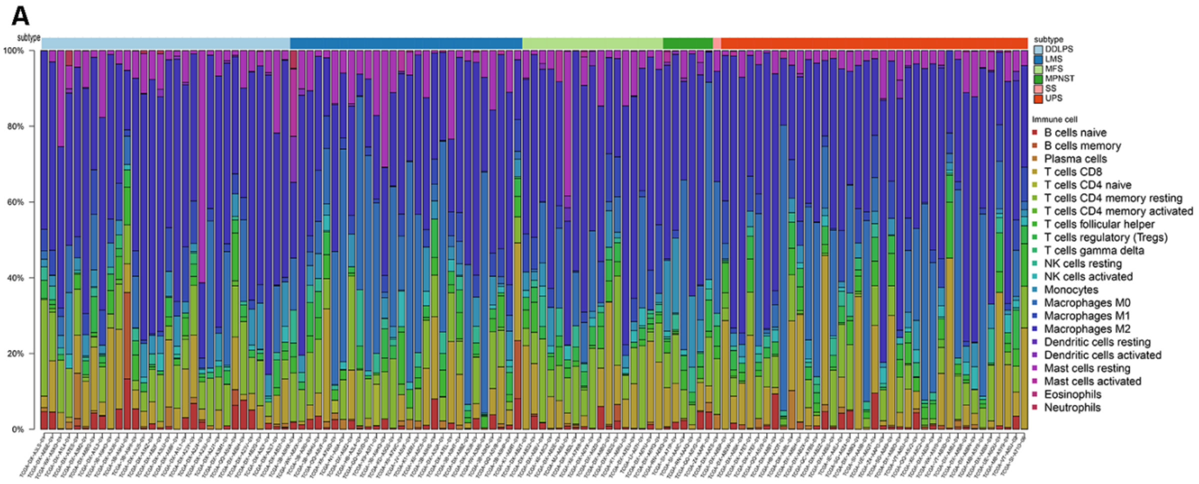

Supplementary Figure 1. Infiltration of immune cell subpopulations in sarcoma samples. (**A**) Stacked bar chart of immune cells estimated by the CIBERSORT algorithm in sarcomas samples.

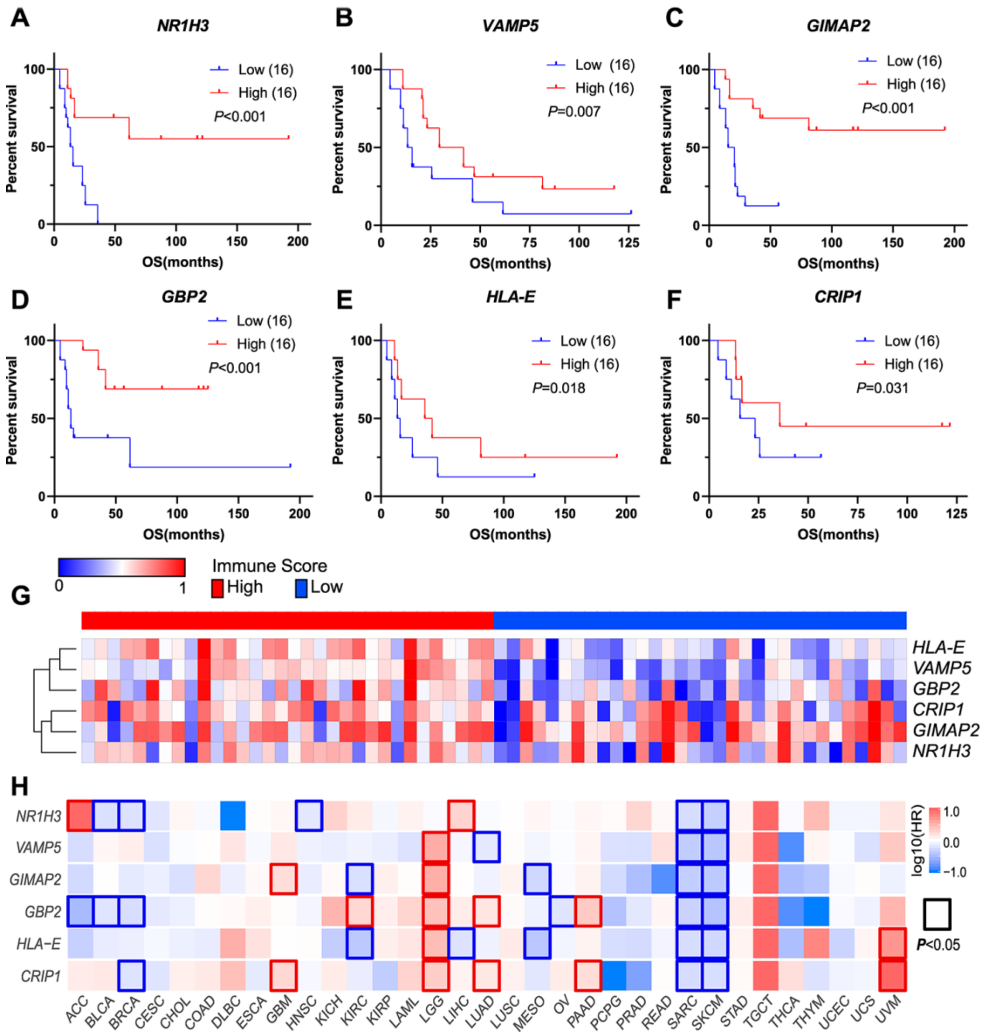

Supplementary Figure 2. Validation of the DEG correlation extracted from the database of TCGA with overall survival in GSE17679. The Kaplan-Meier survival curves for sarcoma patients further separated into high and low expression groups based on the quartiles of NR1H3 (**A**), VAMP5 (**B**), GIMAP2 (**C**), GBP2 (**D**), HLA-E (**E**) and CRIP1 (**F**), separately. (**G**) Heatmap of the mRNA levels of prognosis-related genes in dataset GSE17679. (**H**) The Mantel-Cox tests compare the survival contribution of NR1H3, VAMP5, GIMAP2, GBP2, HLA-E and CRIP1 mRNA levels (the top 30% and the bottom 30%, respectively) in patients from 33 cancer cohorts. The HR was restricted to the range −10 to 10 by replacing all values > 10 with 10 and all values < −10 with −10. HR: Hazard ratio.

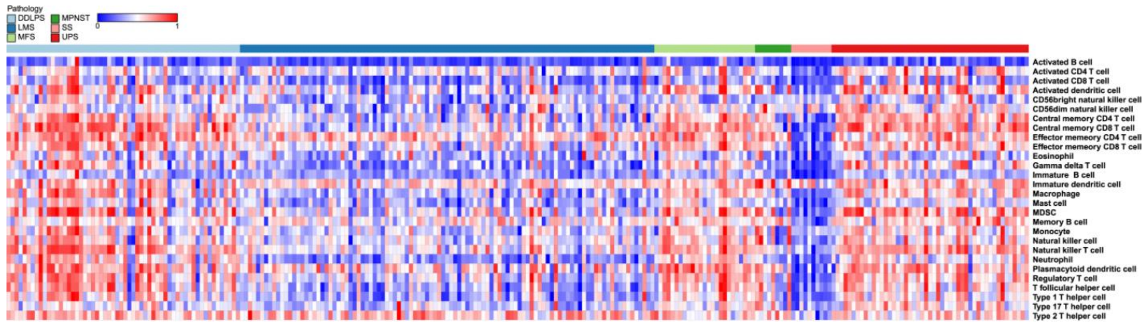

Supplementary Figure 3. Scores of immune cell subpopulations in sarcoma samples. Single-sample gene set enrichment analysis identified the relative infiltration of immune cell subpopulations for 254 sarcoma samples. The relative infiltration of each cell type was normalized by min-max normalization.
