# Supplementary material for: Comprehensive analysis of immune infiltration and gene expression for predicting survival in patients with sarcomas
Source: Aging (Albany NY). 2020 Dec 9;13(2):2168–83. doi: 10.18632/aging.202229 (PMC7880383; doi:10.18632/aging.202229)
Supplement: Supplementary Table 1 [file aging-13-202229-s002.pdf]

SUPPLEMENTARY TABLE

Supplementary Table 1. Genes associated with good prognoses identified in TCGA.

**Interferon-γ-mediated signaling pathway**	CIITA, ICAM1, HLA-DQB2, HLA-A, HLA-C, OAS1, HLA-B, OAS2, HLA-E, TRIM22, HLA-DQA2, HLA-F, B2M, OASL, CD44, IRF5, IFNG, IRF8, IRF1, HLA-DPA1, HLA-DPB1, GBP2, GBP1, HLA-DRA
**Antigen processing and presentation via MHC class II**	HLA-DQB2, HLA-DPA1, HLA-DPB1, HLA-DMA, HLA-DQA2, HLA-DOB, HLA-DRA
**Antigen processing and presentation via MHC class I**	TAP1, HLA-A, HLA-C, HLA-B, HLA-E, TAPBP, HLA-F, B2M
**Macrophage chemotaxis**	CCL2, LGALS3, CCL5
**Neutrophil chemotaxis**	CCL2, LGALS3, S100A9, CCL8, CCL5, VAV1, CCL18, CCL17, CCL22, CCL13, CCL23, IFNG, CSF3R, XCL1, XCL2, SYK
**Dendritic cell chemotaxis**	CCR7, CCR5, CCR2, CCL19, CCL5
**Positive regulation of T cell chemotaxis**	CCR2, TNFSF14, CCL5, XCL1
**B cell activation**	IKZF3, CXCR5, LAX1, ZAP70, CD79A, CD40, PRKCB, BTK
